# Integrated Navigation System Design for Micro Planetary Rovers: Comparison of Absolute Heading Estimation Algorithms and Nonlinear Filtering

**DOI:** 10.3390/s16050749

**Published:** 2016-05-23

**Authors:** Muhammad Ilyas, Beomjin Hong, Kuk Cho, Seung-Ho Baeg, Sangdeok Park

**Affiliations:** 1 Department of Robotics and Virtual Engineering, Korea University of Science and Technology (UST), Daejon 305-333, Korea; milyasmeo@kitech.re.kr (M.I.); hbj723@kitech.re.kr (B.H.); 2 Robotics R & BD Group, Korea Institute of Industrial Technology (KITECH), Ansan 426-791, Korea; googi33@kitech.re.kr (K.C.); sdpark@kitech.re.kr (S.P.)

**Keywords:** planetary rovers navigation, absolute heading angle estimation, multi-sensor fusion, Sun sensor, Extended Kalman filter, Unscented Kalman filter

## Abstract

This paper provides algorithms to fuse relative and absolute microelectromechanical systems (MEMS) navigation sensors, suitable for micro planetary rovers, to provide a more accurate estimation of navigation information, specifically, attitude and position. Planetary rovers have extremely slow speed (~1 cm/s) and lack conventional navigation sensors/systems, hence the general methods of terrestrial navigation may not be applicable to these applications. While relative attitude and position can be tracked in a way similar to those for ground robots, absolute navigation information is hard to achieve on a remote celestial body, like Moon or Mars, in contrast to terrestrial applications. In this study, two absolute attitude estimation algorithms were developed and compared for accuracy and robustness. The estimated absolute attitude was fused with the relative attitude sensors in a framework of nonlinear filters. The nonlinear Extended Kalman filter (EKF) and Unscented Kalman filter (UKF) were compared in pursuit of better accuracy and reliability in this nonlinear estimation problem, using only on-board low cost MEMS sensors. Experimental results confirmed the viability of the proposed algorithms and the sensor suite, for low cost and low weight micro planetary rovers. It is demonstrated that integrating the relative and absolute navigation MEMS sensors reduces the navigation errors to the desired level.

## 1. Introduction

Planetary rovers are wheeled robots characterized by extremely slow speed and rotation. They typically move in a stop-and-go fashion so that they can scan their surroundings to find a drivable path, and discover scientific targets in the exploration area. For example, NASA’s Mars Exploration Rovers have an average speed of 1 cm/s when moving on hard and flat surfaces and even less in normal motion [1]. Typical motion scenarios of planetary rovers include straight drives, arc turns and turn-in-place maneuvers, *etc.* [2].

To explore an unknown environment, the rover must know two things: “where am I?” and at the same time, “where am I heading to?” The answers to these questions are essential to allow the manned/unmanned robot to perform reliably and not become lost in a given environment. Navigation would appear to be easy for planetary rovers due to their slow dynamics, but unfortunately the sensors that are available for terrestrial robot navigation are not always available onboard the planetary rovers. In terrestrial applications the traditional methods of estimating the attitude and position of mobile robots include dead reckoning, use of a global positioning system (GPS), magnetic compass and map matching, *etc*. However, none of these methods alone can solve the problem of self-localization on another planet. For example, a GPS-like system is not available on the Moon or Mars yet. Magnetic compasses commonly employed for absolute heading estimations on Earth cannot be used on other planets. For instance, the magnetic field on Mars and Moon is too weak and irregular to be useful as a navigational aid [3,4]. Similarly, gyro-compassing, which is a common method of seeking the north by high-grade IMUs using Earth’s rotation [5], is not suitable on many other planets, due, for example, to the extremely slow rotation of the Moon around its own axis. Digital maps are not available or have too low resolution to provide accurate navigation information for planetary rovers. All these limitations ultimately make planetary rovers navigation in a completely unknown environment difficult. Therefore, there is an urgent need to solve the dual problems of relative and absolute navigation on remote planets.

Relative (local) navigation information can be obtained in a way similar to that used for terrestrial ground vehicles, e.g., through integration of angular rates to get attitude and displacement from wheel encoders [6]. However, absolute (global) navigation information, which provides the navigational information of a vehicle directly in global coordinates and does not diverge with the passage of time, is difficult to obtain on a planetary surface. Another reason that absolute navigational information is needed in the navigating framework of a planetary rover is to limit the unbounded error growth of a dead reckoning positioning system. Absolute heading is especially essential to reduce cross track errors in mobile robots [7].

The field of planetary rover navigation has been widely researched since the launch of the Path-finder mission by NASA in 1997. A Sun sensor was developed in [8] to provide planetary rovers with heading estimations, but the algorithm was computationally too burdensome to be used on actual rovers. Further improvements in design and usage were provided in [9]. A charge-coupled device (CCD) type sensor was used in [10] which has a wide FOV and was modeled as a fish-eye lens. It involves a complex calibration procedure with 21 parameters. An algorithm to estimate absolute heading and relative position using a Sun sensor, Earth sensor and an inclinometer was provided in [11]. The attitude estimation algorithm was described from multiple vector observations using optimization based on Davenport’s *q-Method* [12] However, the authors validate their propositions only through simulations, and provide no real data from field experimentation. They also do not consider all possible motion scenarios, as was done in the present study.

A position sensitive detector (PSD) type Sun sensor, similar to ours, has been used in [13]. The PSD type Sun sensor uses less power and simple algorithm to detect the Sun’s position in the sensor frame, and suffers less atmospheric effects and is quite robust. The authors also provided an algorithm for estimating absolute heading and absolute position using the Sun sensor and an inclinometer, employing the *q-Method*. The accuracy of position estimation was quite coarse. They achieved absolute heading angle within a few degrees but used a nonlinear minimization approach, which requires much more time as compared to our approach. Furthermore, the optimization based methods can only be employed in a static condition [14], and requires the vehicle to stop for few minutes [15] during the collection of the sensor’s multiple observations. In contrast, our approach is a *one-shot* heading estimation method, called Estimation of absolute Attitude using Sun sensor and Inclinometer (*EASI*), and can be performed in real time at 10 Hz (determined by the Sun sensor’s update rate), using the Sun sensor and inclinometer data only.

The Sun is the most prominent navigation beacon for space applications. We have designed an absolute heading estimation algorithm which employs low cost, low power and low volume MEMS sensors, suitable for micro rovers (<20 kg). Efforts to employ such miniaturized navigation sensors are already occurring [16,17]. Further, to achieve reliable and more accurate navigation information at a high data rate, we have integrated the relative and absolute navigation sensors in a framework of nonlinear filters. We only chose and compared non-linear filters which can be executed with the limited computation resources of micro planetary rovers, e.g., the Extended Kalman filter (EKF), Unscented Kalman filters (UKF), *etc.* Other non-linear filters which have high computational expense (e.g., Particle filters, Divided difference filters, *etc.*) are not considered in this work, for the sake of simplicity and applicability.

The aim of this work is to achieve reliable and accurate navigation estimation within the desired goals of a planetary rover, e.g., with less than 10% error in position and 2° in attitude [18], taking into account all possible motion scenarios, including long time stationary, move in straight and curved paths, and take turn-in-place (*TiP)* motion [2], *etc.*

The following are the main elements of this study. First we provide an algorithm (*EASI*) to estimate the absolute heading angle using MEMS sensors, specifically, a Sun sensor and inclinometer. We integrate the relative and absolute navigation sensors to achieve an improvement in navigation accuracy and reliability. Our integration filter integrates the gyro, Sun sensor and inclinometer for long term attitude accuracy, and hence stabilizes the estimation of position. When the rover stops, we use the Sun sensor and inclinometer data to estimate absolute heading and roll, pitch angles. During in-motion state, the integration filter fuses data from the gyro, Sun sensor, and inclinometer to continuously track the attitude. Drifts in gyro-only attitude are bounded by the absolute information of the *EASI* algorithm. Next, the optimization based heading estimation algorithm (*q-Method*) and *EASI* algorithm are compared in different motion scenarios, and finally the nonlinear fusion filters (EKF/UKF) are compared in terms of accuracy and reliability. The aim is to show the viability of using minimal/low-cost MEMS sensors in a micro rover navigation system having very limited space and power resources.

**Figure 1:**
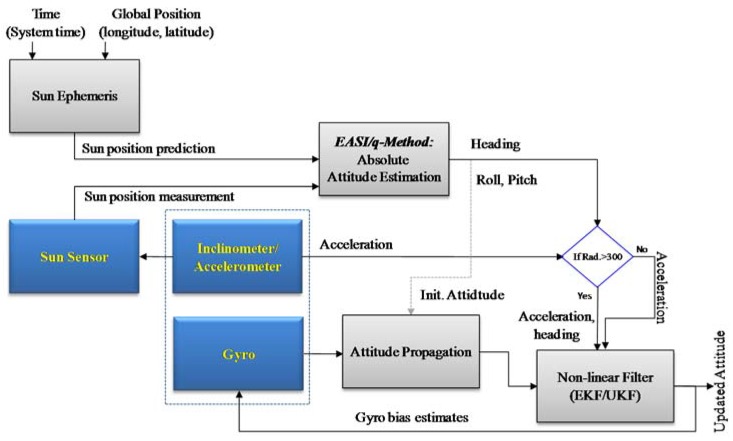
Algorithm block diagram for integrating relative and absolute navigation sensors for attitude estimation.

The conceptual diagram of the relative and absolute sensors integration algorithm is shown in Figure 1. At the end of this work, we will show the benefits gained by integrating the relative and absolute navigation sensor data, and compare two nonlinear filters, using real experimental data.

The organization of this paper is as follows: in Section 2, we present the attitude initialization and propagation algorithm, and in Section 3, two absolute attitude estimation algorithms are provided in detail. Section 4 deals with multi-sensor fusion and description of the hardware used in this work. In Section 5, we present the experimental setup, comparisons of the results and discussion. And finally in Section 6, we conclude this paper with some comments on future research directions.

## 2. Attitude Estimation in Planetary Environment

Keeping track of attitude is important to ensure accurate localization; heading accuracy is especially effective at reducing cross track errors and hence enhances the accuracy of overall localization [19]. Although gyroscopes can be used for such purpose, attitude propagation using only gyros requires mathematical integration of the angular rate signals obtained from the sensor. This causes the noise in the gyro signal to be integrated as well, along with the actual angular rate signal. Moreover, the gyro bias drift will contribute to calculated attitude divergence with time propagation. This is the inherent limitation of gyro-only attitude estimation, and occurs no matter how accurate and expensive a gyro sensor is used. To address this task, first we have to define the different coordinate frames which are used to represent navigation information in this work.

### 2.1. Coordinate Frames

**Figure 2:**
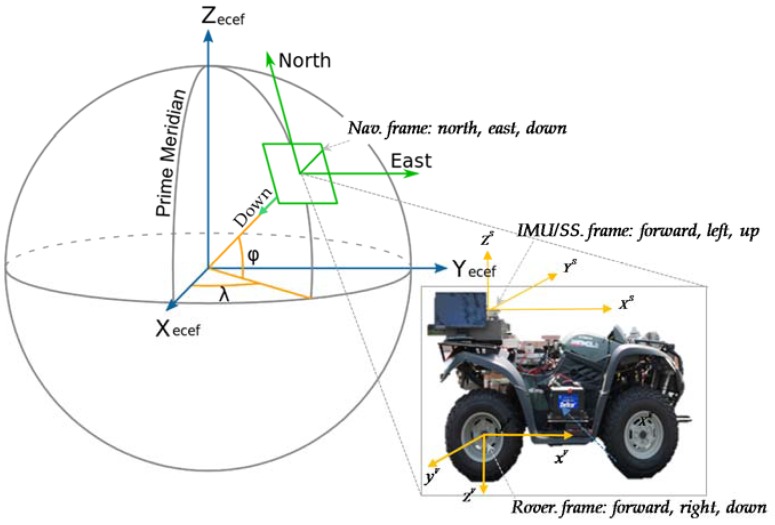
Illustration of the reference frames used for Planetary Rovers.

Different sensors are located with different orientations and provide observations in their respective coordinates. In order to use vector observations in a common frame to produce meaningful results, one has to perform transformations among the different coordinate frames. In this work, we have defined the coordinate frames in perspective of planetary rover’s navigation, as shown in Figure 2. The navigation frame (n-frame) is most conventionally defined as North-East-Down (NED) frame, IMU and Sun sensor frames are defined in forward, left and up directions. However, the rover frame corresponds to the definition of aerospace vehicles, e.g., by forward, right and down axes. Transformation matrices used in this work are given in our previous work [20].

### 2.2. Attitude Initialization

For a planetary rover, the tilt angles (*i.e.*, roll, pitch) may be initialized by an inclinometer-based angle estimation in a static or quasi-static condition. Since it is not possible to use magnetic or gyro-compasses on most of the other planets, estimating absolute heading is more challenging in a planetary environment. We present an algorithm that uses the Sun sensor and inclinometer data to estimate absolute heading, and then initialize our navigation algorithm with the heading estimated by this algorithm.

#### 2.2.1. Initialization of Roll and Pitch Angles using Inclinometer

In a static condition an inclinometer senses gravitational acceleration and hence can be used to calculate tilt angles. As gravity in a local tangent n-frame can be transformed, using transformation matrix $C_{n}^{b}$, to a b-frame by compensating with roll (ϕ), pitch (θ) and yaw (ψ) angles, Equation (1) gives the gravity vector in the b-frame. Since the same quantity is measured by the inclinometer in the b-frame, we can calculate the roll and pitch angles, Equations (2) and (3), between these two frames [21] by comparing the L.H.S and R.H.S of Equation (1), where M samples of the inclinometer data are averaged over the stationary time period.

(1) $$\left(\begin{array}{c} f_{ib,x}^{b}\\ f_{ib,y}^{b}\\ f_{ib,z}^{b} \end{array}\right)=g^{b}=C_{n}^{b}g^{n};\;∴C_{n}^{b}=[\begin{array}{ccc} c\;\theta c\psi & c\;\theta s\psi & -s\;\theta \\ -c\phi s\psi +s\phi s\theta c\psi & c\phi c\psi +s\phi s\theta s\psi & s\theta c\phi \\ s\phi s\psi +s\phi s\theta c\psi & -s\phi c\psi +c\phi s\theta s\psi & c\phi c\theta \end{array}]\;∴C_{n}^{b}\in {\mathbb{R}}^{3\times 3},g^{n}\in {\mathbb{R}}^{3\times 1};\;c(.)\equiv \cos(.),s(.)\equiv \sin(.)$$

(2) $$Roll\equiv \phi =\tan^{-1}\left(\frac{{\bar{f}}_{ib,y}^{b}}{{\bar{f}}_{ib,z}^{b}}\right)$$

(3) $$Pitch\equiv \theta =\tan^{-1}\left(\frac{{\bar{f}}_{ib,x}^{b}}{\sqrt{{\left({\bar{f}}_{ib,y}^{b}\right)}^{2}+{\left({\bar{f}}_{ib,z}^{b}\right)}^{2}}}\right);\;∴\;{\bar{f}}_{ib,xyz}^{b}=\frac{1}{M}\sum_{j=1}^{M}{\bar{f}}_{ib,j}^{b}$$

#### 2.2.2. Initialization of Heading Angle Using Sun Sensor and Inclinometer

As mentioned previously, obtaining absolute heading angle with respect to true north on a remote planet surface is a hard problem, due to the lack of conventional heading sensors that are available on Earth or near the Earth’s surface. Sun observations have long been used for navigational purposes because the Sun is a very reliable navigational beacon, especially in a planetary environment where there is no atmospheric interference. We initialize our heading estimate using the Sun sensor and inclinometer data (*i.e.*, with the *EASI* algorithm or *q-Method*). Details of these algorithms are provided in Section 4.

### 2.3. Relative Attitude Estimation Using IMU

There are several methods for expressing the orientation of objects relative to some reference. However, the three most common approaches used are: Euler angles, Direction cosines matrix (DCM) and Quaternion based attitude representation. Euler angles have 12 different formulations, so their sequence of rotation must be defined first. Moreover Euler angles have mathematical singularity (Gimbal lock) that exists when the pitch angle approaches 90°. But for most of the practical platforms in use, especially for planetary rovers, as in this work, this poses no real threat. Positive aspect of Euler angles is the common familiarization of attitude representation with roll, pitch and yaw angles. The DCM is complex to use but is stable. It involves nine elements to be estimated. Quaternions are simplest to use and have no singularities. The only downside is that quaternions are regarded as mysterious and conceptually difficult to understand.

The Euler angle can represent the attitude of a platform from three successive rotations of the navigation frame to the rover frame. In this work, the navigation frame is rotated and fitted into the rover frame in the sequence of yaw, pitch and roll. The transformation of the gyro sensed angular velocity to the Euler angular velocity can be achieved by the following relation [22]:

(4) $$\begin{array}{c} {\dot{\Psi }}_{k}=\left[\begin{array}{c} \dot{\phi }\\ \dot{\theta }\\ \dot{\psi } \end{array}\right]=\left[\begin{array}{ccc} 1 & \sin(\phi )\tan(\theta ) & \cos(\phi )\tan(\theta )\\ 0 & \cos(\phi ) & -\sin(\phi )\\ 0 & \sin(\phi )/\cos(\theta ) & \cos(\phi )/\cos(\theta ) \end{array}\right]\left[\begin{array}{c} \omega_{x}\\ \omega_{y}\\ \omega_{z} \end{array}\right]\\ {\dot{\Psi }}_{k}=\left[E_{b}^{n}\right]\omega_{ib}^{b} \end{array}$$

where Ψ represents attitude vector and $\omega_{ib}^{b}={\left[\omega_{x}\;\omega_{y}\;\omega_{z}\right]}^{T}$ is the gyro output and matrix $E_{b}^{n}$ relates the rover body angular velocity to the rate of change of Euler angles. Note that the solution of Equation (4) requires the integration of the first-order nonlinear equation. In discrete time, attitude propagation can be done by simple numerical integration, *i.e.*

(5) $$\left[\begin{array}{c} \phi_{k}\\ \theta_{k}\\ \psi_{k} \end{array}\right]=\left[\begin{array}{c} \phi_{k-1}\\ \theta_{k-1}\\ \psi_{k-1} \end{array}\right]+\int_{k-1}^{k}\left[\begin{array}{c} {\dot{\phi }}_{k}\\ {\dot{\theta }}_{k}\\ {\dot{\psi }}_{k} \end{array}\right]\;dt$$

(6) $$\Psi_{k}=\Psi_{k-1}+{\dot{\Psi }}_{k}\Delta t\;;\Psi_{k}\in {\mathbb{R}}^{3\times 1}$$

## 3. Absolute Attitude Update Using Sun Sensor and Inclinometer in a Planetary Environment

Gyroscopic-based attitude propagation suffers from divergence with time due to the presence of noise in the sensor signals, and consequently this method cannot be used for estimating attitude for operations over a long period of time. The unbounded growth in error in attitude that occurs when it is solely determined by gyroscope must be limited by some absolute reference; otherwise the estimation will become useless after some time, depending on the accuracy of the gyros used.

In near-Earth applications, we have many options for obtaining the absolute heading of the vehicle, but there is a limited number of heading estimation sensors in a planetary rover application. The Sun is the most prominent navigation beacon in the sky and as the technology develops, use of the Sun for navigating unmanned vehicles in space is becoming more practical.

In this section we briefly present two absolute heading estimation algorithms:(i) the Estimation of Absolute heading using a Sun sensor and Inclinometer (*EASI*), developed in our previous work [20]; and (ii) an optimization-based algorithm (*q-Method*) [11,13]. Both algorithms incorporate information from a Sun sensor and an inclinometer, and produce the sensor’s absolute roll, pitch angles and heading angle with respect to true north at a particular instant in time, date and location on the surface of the planet during operation.

### 3.1. The Sun Position Tracking and Sun Ephemeris

**Figure 3:**
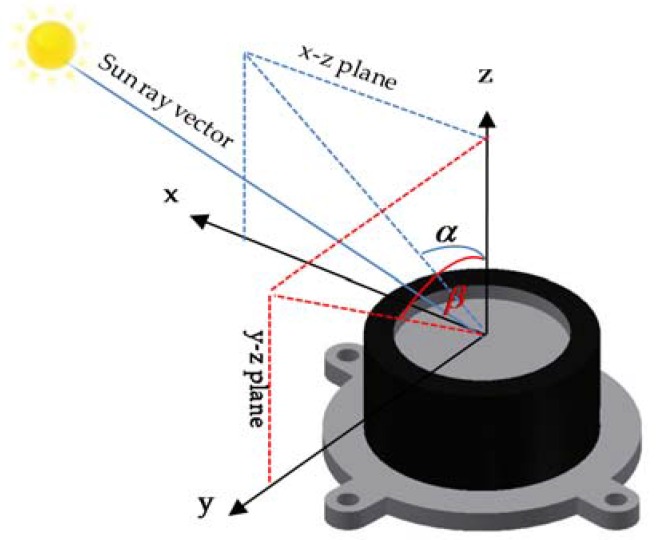
CAD diagram of a PSD type Sun sensor’s (ISS-D60) reference frame and output angles.

Absolute heading estimation algorithms rely on accurate tracking of the Sun’s position by some optical sensor, such as a CCD image sensor or a PSD type sensor, which measures the position of a light spot in one or two dimensions on the sensor’s surface. Using any kind of optical sensor, the goal is to track the Sun position, *i.e.*, the Sun’s azimuth and elevation angles, as it sweeps across the horizon. The Sun ephemeris is a tabulated dataset based on mathematical models of the motion of the Sun and Earth. Given a date and time, these tables also provide a very precise Sun position in the inertial frame. After some transformations, the Sun position can be predicted in the navigation frame [23].The PSD type Sun sensors, as used in this work and shown in Figure 3, measure the incidence angle of a Sun ray in both azimuth and elevation based on a quadrant photodetector device.

The sunlight is guided to the detector through a window above the sensor. Depending on the angle of incidence, the sunlight induces photocurrents in the four quadrants of the detector. The angle α is an angle between the *z*-axis and the projection of the Sun ray on the *x*-*z* plane. Similarly, the angle β is an angle between the *z*-axis and the projection of the Sun ray on the *y*-*z* plane. These two angles are also referred to as Angle-*x* and Angle-*y*, respectively, in the Sensor’s data sheet [24].

### 3.2. Estimation of Absolute Heading Using Sun Sensor and Inclinometer (EASI Algorithm)

**Figure 4:**
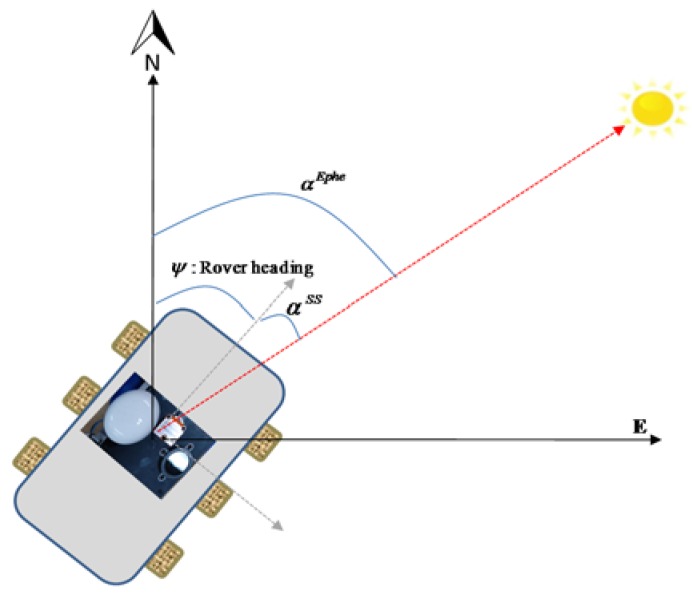
Illustration of the relation between rover heading angle obtained from the Sun position using the Sun sensor, and from Sun ephemeris data. *α^Ephe^* is the Sun’s azimuth angle from the ephemeris data, ***α^SS^*** is the Sun azimuth angle from the Sun sensor and $\psi_{ss}$ is the rover’s heading angle [20].

The Sun sensor used in this work does not directly give the Sun’s position. However, the Sun’s azimuth and elevation angles can be calculated from the sensor’s output angles (*i.e.*, angle *α*, angle *β*) using simple trigonometry. The mathematical details of the *EASI* algorithm are given in our previous work [20]. The *EASI* algorithm is described here briefly in Algorithm 1, below. The relation between the Sun’s position and the rover’s absolute heading angle is depicted in Figure 4.
**Algorithm 1.** Estimation of Absolute heading angle using Sun sensor and Inclinometer (*EASI* algorithm)Get Sun ray vector from sensor output angles.$S^{s}={\left[\begin{array}{ccc} S_{x} & S_{y} & S_{z} \end{array}\right]}^{T}$Transform **S***^S^* to rover frame.$S^{b}=\left[\begin{array}{c} S_{x}^{b}\\ S_{y}^{b}\\ S_{z}^{b} \end{array}\right]=C_{s}^{b}\left[\begin{array}{c} S_{x}\\ S_{y}\\ S_{z} \end{array}\right]∴C_{s}^{b}=\left[\begin{array}{ccc} 1 & 0 & 0\\ 0 & -1 & 0\\ 0 & 0 & -1 \end{array}\right]$Transform **S***^b^* to local-flat frame.$S^{n}=\left[\begin{array}{c} S_{x}^{n}\\ S_{y}^{n}\\ S_{z}^{n} \end{array}\right]=C_{b}^{n}\left[\begin{array}{c} S_{x}^{b}\\ S_{y}^{b}\\ S_{z}^{b} \end{array}\right]\;∴\;C_{b}^{n}=\left[\begin{array}{ccc} \cos\theta & \sin\phi \sin\theta & \cos\phi \sin\theta \\ 0 & \cos\phi & -\sin\theta \\ -\sin\theta & \sin\phi \cos\theta & \cos\phi \cos\theta \end{array}\right]$Get Sun’s azimuth (*α^SS^*) and elevation angles ($\varsigma^{ss}$) from $S^{n}$ vector as:$\begin{array}{l} \alpha^{ss}=a\tan2(S_{y}^{n^{\prime }},S_{x}^{n^{\prime }})\\ \varsigma^{ss}=-\sin^{-1}(S_{z}^{n^{\prime }}) \end{array}$Using local date, time and location (latitude, longitude), get Sun’s azimuth ($\alpha^{Ephe.}$) and elevation ($\varsigma^{Ephe.}$) angles from Ephemeris models.Determine rover’s absolute heading by comparing the Sun’s position in steps 4 and 5.$\psi_{ss}=\left\{ \begin{array}{l} \alpha^{Ephe}-\alpha^{SS}\;if(\alpha^{Ephe}>\alpha^{SS})\\ \alpha^{SS}-\alpha^{Ephe}\;otherwise \end{array}\right.$

### 3.3. Optimization-Based Absolute Attitude Estimation (q-Method)

Optimization based heading estimation is another method for estimating absolute roll, pitch and heading angles based on multiple Sun observations and gravity vector observations. It uses at least a pair of Sun and gravity vectors and applies optimization techniques to minimize the cost function. However, in practice many observations must be collected from both sensors before applying optimization technique. The result is a transformation matrix between two observation frames, in this case, the Planet-centered Planet-fixed frame (F-frame) and the Sun sensor frame (S-frame). Using this approach, as explained in [11,15] we obtain prediction vectors, namely the Sun position and gravity vector, in the F-frame from the Sun ephemeris data, and from the gravity model, respectively, as in Equation (7):

(7) $$\begin{array}{l} S^{F}=C_{I}^{F}S^{I}\\ g^{F}=C_{n}^{F}g^{n} \end{array}$$

where **S***^I^* and **g***^n^* are vectors in the inertial frame (I-frame) and n-frame. The corresponding measurements of the Sun position ($S^{s}$) and gravity vector ($g^{s}$) are obtained from the Sun sensor and inclinometer in the sensor frame. Note that the transformation between the inclinometer and Sun sensor frame is assumed to be a unity matrix, *i.e.*, both sensors are assumed to be perfectly aligned. After we capture the predicted and measured vectors in two different frames, it simply becomes Wahba’s attitude estimation problem based on vector observations [25]. The aim is to minimize the cost function, which relates two sets of observation vectors (V,W), *i.e.*,:

(8) $$J(C_{F}^{S})=\frac{1}{2}\sum_{i=1}^{m}a_{i}{\left\|V-C_{F}^{S}W\right\|}^{2}$$

Many methods are available to estimate attitude from a multiple vector observation, e.g., QUEST, FOAM, Davenport’s *q-Method* [12], *etc.* By applying any of these methods, we can get the rotations matrix between two observation frames. In addition, this rotation matrix is used to find the absolute tilt angles of the rover with respect to a local level frame, and heading angle from true north.

**Figure 5:**
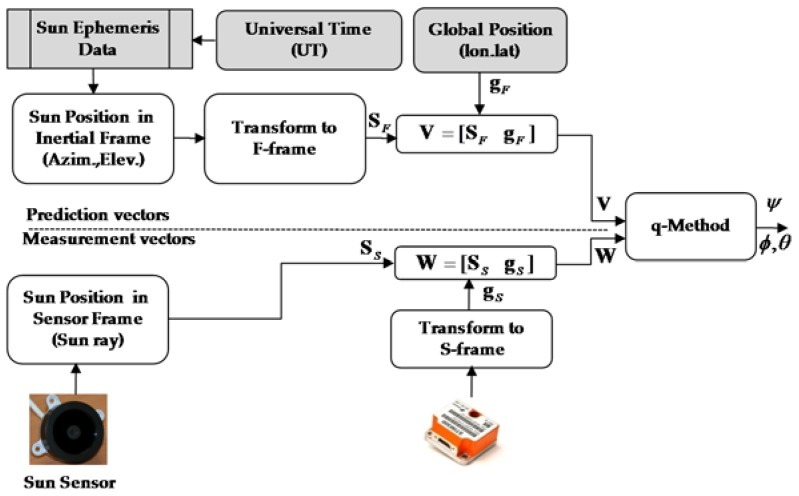
Optimization based attitude estimation (*q-Method*) algorithm block diagram.

To start with, we concatenate the predicted and observed vectors and apply Davenport’s *q-Method* for rotation estimation. This method is explained with mathematical details in [15]. The output of this rotation estimation algorithm is a matrix ($C_{F}^{S}$) which describes the rotation between two observation frames. Figure 5 and Algorithm 2 show the block diagram and mathematical flow of *q-Method*.

**Algorithm 2.** Optimization based absolute attitude estimation algorithm (*q-Method*)Concatenate observation vectors:$\begin{array}{l} W=[S_{S}\;g_{S}]\;∴measurement\;vectors\;in\text{ S-}frame\\ V=[S_{F}\;g_{F}]\;∴preditction\;vectors\;in\text{ F-}frame \end{array}$Calculate: **B** = **WV***^T^*; **Q** = **B** + **B***^T^*Extract **Z** from **B** matrix: **Z** = [*B*_23_ − *B*_32_*B*_31_ − B_13_*B*_12_ − *B*_21_]Form 4 × 4 matrix **K**: $K_{4\times 4}=\left[\begin{array}{cc} Q-I\sigma & Z^{T}\\ Z & \sigma \end{array}\right]\;∴\sigma =tr(B),I=I_{3\times 3}$Find Eigen data of **K**. The Eigenvector corresponding to the largest Eigen value of K is **q** vector:$q_{{}_{4\times 1}}=\left[\begin{array}{c} q_{v}\\ q_{s} \end{array}\right];\;4\times \text{1 unit quaternion representing best fit rotation from}\;F\text{ to }S.$Desired rotation matrix can be found from quaternion vector:$\begin{array}{l} C_{F}^{S}=(q_{s}^{2}-q_{v}^{T}q_{v})I+2q_{v}q_{v}^{T}-2q_{s}q_{v}^{\times }\;∴q_{v}^{\times }=\left[\begin{array}{ccc} 0 & -q_{v_{3}} & q_{v_{2}}\\ q_{v_{3}} & 0 & -q_{v_{1}}\\ -q_{v_{2}} & q_{v_{1}} & 0 \end{array}\right]\\ q_{v}^{\times }:skew-symmetric\;matrix\;form\;of\;q_{v}. \end{array}$Attitude angles extraction:$\begin{array}{l} C_{S}^{T}=C_{F}^{T}C_{S}^{F},\text{a transformation matrix (function of RPY) from b-frame to n-frame}.\\ ∴C_{F}^{T}\;is\;function\;of\;longitude,latitude(rover\;position). \end{array}$Hence roll, pitch and heading angles can be derived from matrix ($C_{S}^{T}$) as:$\begin{array}{l} \phi =a\sin(C_{S}^{T}{}_{32},C_{S}^{T}{}_{33})\\ \theta =a\sin(C_{S}^{T}{}_{31})\\ \psi =a\tan2(C_{S}^{T}{}_{21},C_{S}^{T}{}_{11}) \end{array}$

## 4. Multi-Sensor Data Fusion: Design and Comparison of Non-linear Filters

The attitude of the rover is propagated as given in Equations (5) in Section 2.3. Two nonlinear filters, the Extended Kalman filter and Unscented Kalman filter, were designed and compared in pursuit of better accuracy in this nonlinear environment. In designing EKF and UKF, we assumed low dynamic acceleration of the planetary rovers, which is a valid assumption for slow speed planetary rovers, and utilized the acceleration data from the inclinometer/accelerometer directly in the measurement equation. Hence both the process model and measurement model are nonlinear, involving sinusoidal terms. First, we present the predicted and updated parts of the EKF and UKF filters.

### 4.1. Extended Kalman Filter and Unscented Kalman Filter Algorithm

#### 4.1.1. Process Model

We choose the state vector as follows:

(9) $$x_{k}={\left[\Psi \;b_{xyz}^{g}\right]}^{T}$$

where $\Psi ={\left[\phi \;\theta \;\psi \right]}^{T}$ are roll, pitch, yaw angles and $b_{xyz}^{g}$ represents the three gyro bias components. The discrete time nonlinear process model to be used for attitude propagation in EKF/UKF is given below:

(10) $$x_{k+1}=f(x_{k},u_{k})+w_{k};\;w_{k}\sim \mathbb{N}(0,Q_{k})$$

where, $x_{k}\in {\mathbb{R}}^{n}$ is n×1 state vector, $w_{k}\in {\mathbb{R}}^{q}$ is q×1 state noise process vector, with covariance matrix $Q_{k}$. The system Jacobean matrices, which are used in the EKF algorithm, are computed as:

(11) $$F_{k}={\frac{\partial f_{k}}{\partial x}|}_{{\hat{x}}_{k}^{+}};G_{k}={\frac{\partial f_{k}}{\partial w}|}_{{\hat{x}}_{k}^{+}}$$

Then, the time prediction step in EKF is:

(12) $${\hat{x}}_{k+1}^{-}=f_{k}({\hat{x}}_{k}^{+},u_{k})\;P_{k+1}^{-}=F_{k}P_{k}^{+}F_{k}^{T}+G_{k}Q_{k}G_{k}^{T}$$

The time prediction step in UKF does not require calculation of the Jacobean matrices, instead it generates sigma points $\left({\hat{x}}_{k}^{\left(i\right)}\right)$ based on the a priori state estimate and associated covariance matrix [26]. The UKF algorithm then propagates these sigma points directly through the nonlinear system dynamics, as in Equation (13):

(13) $$\begin{array}{l} {\hat{x}}_{k}^{\left(i\right)}={\hat{x}}_{k}^{+}+\chi^{i},\;i=1,.....,2N\\ ∴\chi^{i}={\left(\sqrt{NP_{k}^{+}}\right)}_{i}^{T},i=1,.....,N\\ ∴\chi^{N+i}=-{\left(\sqrt{NP_{k}^{+}}\right)}_{i}^{T},i=1,.....,N \end{array}\;{\hat{x}}_{k+1}^{\left(i\right)}=f_{k}({\hat{x}}_{k}^{\left(i\right)},u_{k})$$

Then, the transformed sigma points are combined to capture the mean and covariance of the state:

(14) $${\hat{x}}_{k+1}^{-}=\frac{1}{2N}\sum_{i=1}^{2N}{\hat{x}}_{k+1}^{\left(i\right)}\;P_{k+1}^{-}=\frac{1}{2N}\sum_{i=1}^{2N}({\hat{x}}_{k+1}^{\left(i\right)}-{\hat{x}}_{k+1}^{-}){\left({\hat{x}}_{k+1}^{\left(i\right)}-{\hat{x}}_{k+1}^{-}\right)}^{T}+Q_{k}$$

#### 4.1.2. Measurement Update in EKF and UKF

In a measurement update, due to the low dynamics of the planetary rover, it is appropriate to embed the acceleration output directly in the measurement equation. The measurement model, using acceleration from the inclinometer and heading angle calculated in the *EASI*/*q-Method* algorithms, is given below:

(15) $$\left[\begin{array}{c} f_{x}\\ f_{x}\\ f_{x}\\ \psi_{m} \end{array}\right]=\left[\begin{array}{c} C_{n}^{b}g^{n}\\ \psi_{SS} \end{array}\right]+v\;\Rightarrow \left[\begin{array}{c} f_{x}\\ f_{x}\\ f_{x}\\ \psi_{m} \end{array}\right]=\left[\begin{array}{c} -g\sin\theta \\ g\sin\phi \cos\theta \\ g\cos\phi \cos\theta \\ \psi_{ss} \end{array}\right]+v\;y_{k+1}=h(x_{k+1})+v_{k+1};v_{k+1}\sim \mathbb{N}(0,R_{k})$$

where, $y_{k}\in {\mathbb{R}}^{m}$ is m×1observation vector and $v_{k}\in {\mathbb{R}}^{r}$ is r×1 measurement noise process vector, with covariance matrix **R***_k_*. Further it is assumed that: $E\left[w_{k}w_{j}^{T}\right]=\delta_{kj}Q_{k},\;E\left[v_{k}v_{j}^{T}\right]=\delta_{kj}R_{k},\;E\left[v_{k}w_{j}^{T}\right]=0,\;\forall k,j$.

The EKF will require measurement of the Jacobean matrix calculated as below:

(16) $$\begin{array}{l} H_{k+1}={\frac{\partial h}{\partial x}|}_{{\hat{x}}_{k+1}^{-}}=\left[\begin{array}{cc} H_{11} & H_{12}\\ H_{21} & H_{22} \end{array}\right]\\ \text{where},\;H_{11}=\left[\begin{array}{cc} 0 & -g\cos(\theta )\\ g\cos(\phi )\cos(\theta ) & -g\sin(\phi )\sin(\theta )\\ -g\sin(\phi )\cos(\theta ) & g\cos(\phi )\sin(\theta ) \end{array}\right];H_{12}=\left[0_{3\times 4}\right];H_{21}=\left[0_{1\times 2}\right];H_{22}=\left[\begin{array}{cccc} 1 & 0 & 0 & 0 \end{array}\right] \end{array}$$

The measurement update step of EKF proceeds as usual:

(17) $$\begin{array}{l} K_{k+1}=P_{k+1}^{xy}/P_{k+1}^{vv}\\ {\hat{x}}_{k+1}^{+}={\hat{x}}_{k+1}^{-}+K_{k+1}+[y_{k+1}-{\hat{y}}_{k+1}]\\ P_{k+1}^{+}=(I-K_{k+1}H_{k+1})P_{k+1}^{-} \end{array}$$

where, $P_{k+1}^{xy}=P_{k+1}^{-}H_{k+1}^{T}$ and $P_{k+1}^{vv}=H_{k+1}^{}P_{k+1}^{-}H_{k+1}^{T}+R_{k+1}$.

For the measurement update step of UKF, we can calculate sigma points from the freshly predicted state vector and transform the sigma points through the measurement model directly. The transformed sigma points are combined to produce the predicted measurement vector at time k + 1:

(18) $$\begin{array}{l} {\hat{y}}_{k+1}^{\left(i\right)}=h({\hat{x}}_{k}^{\left(i\right)}{}_{k})\\ {\hat{y}}_{k+1}^{-}=\frac{1}{2N}\sum_{i=1}^{2N}{\hat{y}}_{k+1}^{\left(i\right)} \end{array}$$

To update the state and associated covariance matrix, the UKF algorithm proceeds as follows. It first calculates the covariance ($P_{k+1}^{vv}$) of the predicted measurement vector and adds the noise covariance matrix to account for sensor measurement noise. Then, the cross covariance matrix ($P_{k+1}^{xy}$) between the predicted state and predicted measurement vector is calculated as:

(19) $$\begin{array}{l} P_{k+1}^{vv}=\frac{1}{2N}\sum_{i=1}^{2N}({\hat{y}}_{k+1}^{\left(i\right)}-{\hat{y}}_{k+1}){\left({\hat{y}}_{k+1}^{\left(i\right)}-{\hat{y}}_{k+1}\right)}^{T}+R_{k+1}\\ P_{k+1}^{xy}=\frac{1}{2N}\sum_{i=1}^{2N}({\hat{X}}_{k+1}^{\left(i\right)}-{\hat{X}}_{k+1}^{-}){\left({\hat{y}}_{k+1}^{\left(i\right)}-{\hat{y}}_{k+1}\right)}^{T} \end{array}$$

**Figure 6:**
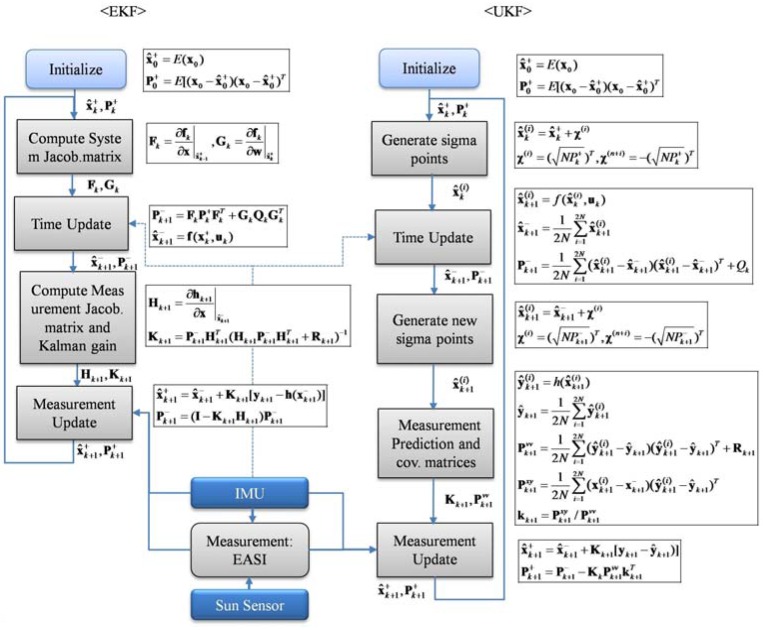
Algorithm flow of Extended Kalman filter and Unscented Kalman filter, with corresponding equations against each processing block.

After this step, the measurement update in UKF takes a form similar to EKF, Equation (17). The whole process flowchart along with the corresponding equations for both nonlinear filters is given in Figure 6.

### 4.2. Position Update

In this work we have calculated the rover position using the traditional *wheel-odometry* method, *gyro-odometry* and by combining the wheel encoder data and the output of the *EASI* algorithm, called *EASI-odometry*. However, even if we provide accurate attitude estimates, the position error will grow due to errors in the dead reckoning process when using wheel encoder data to get change in position, as given in Equations (20) and (21),

(20) $$\left[\begin{array}{c} x_{k}\\ y_{k}\\ z_{k} \end{array}\right]=\left[\begin{array}{c} x_{k-1}\\ y_{k-1}\\ z_{k-1} \end{array}\right]+C_{b}^{n}\left[\begin{array}{c} v_{x}^{wheel}\\ 0\\ 0 \end{array}\right]\Delta t$$

(21) $$P_{k}=P_{k-1}+\Delta P\;;\;P_{k}\in {\mathbb{R}}^{3\times 1}$$

where $v_{x}^{wheel}$ is the forward velocity from wheel encoder and ΔP is the change in position during time Δt. The transformation matrix ($C_{b}^{n}$) can be composed of just the heading angle in the case of wheel-odometry, three propagated attitude angles in the case of gyro-odometry [27] or stabilized attitude in the case of *EASI-odometry*.

For stabilized position estimation (*i.e.*, non-drifting position), we need some external reference to correct the position errors in *PositionEKF*. The *PositionEKF* is designed to update the position only, if position measurements are available from any other external absolute sensor/system. For example, in this work, we have used GPS absolute position measurements (with 5m accuracy) after every 30 s, to simulate absolute position reference data in planetary environment. The measurement model for position updates in *PositionEKF* can be written as:

(22) $$y_{k}=h(P_{k})+\eta_{k}\;;\;\eta_{k}\in \mathbb{R}{\;}^{3\times 1}$$

Note that position measurements must be transformed to local-tangent frame (n-frame) before incorporating in estimator frame work, hence it becomes a non-linear function h(.) with noise vector $\eta_{k}$.

**Figure 7:**
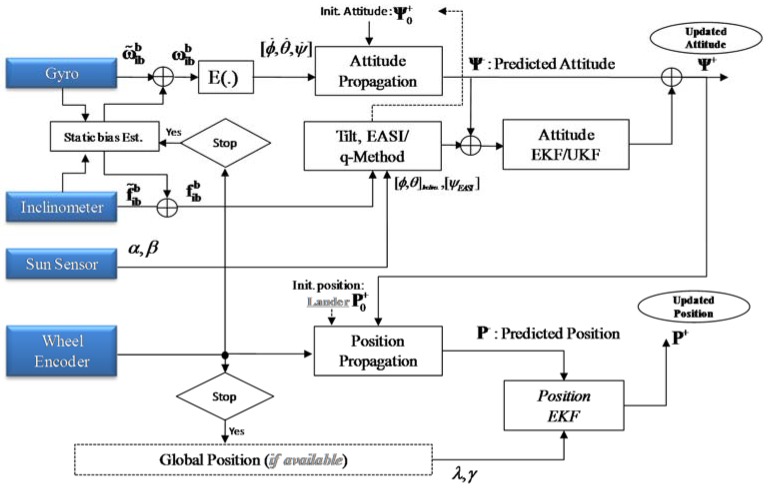
Sensor fusion for planetary rover localization: Block diagram.

This infrequent position measurement is chosen because on a planetary rover, absolute position measurements are not as frequently available as in terrestrial applications [3,28]. Position is propagated using wheel encoder data and attitude estimated from the *EASI* algorithm, and the position is updated if any source of absolute position becomes available, e.g., from the onboard map matching algorithm (DEM), rover tracking from the orbiter circling the planet, direct rover tracking from an Earth-based station, *etc.* None of these external sources of rover position estimation can provide frequent position measurements to update the localization data in *PositionEKF* due to their limitations. However, infrequent position measurements are sufficient if we have precise and well calibrated wheel encoders and there is no wheel slippage or rover skidding during motion. The divergence in localization due to odometry errors may be reduced by incorporating global position information in a position EKF framework, as shown in Figure 7.

### 4.3. Hardware Description of Navigation Sensor Module for Micro Planetary Rover

**Figure 8:**
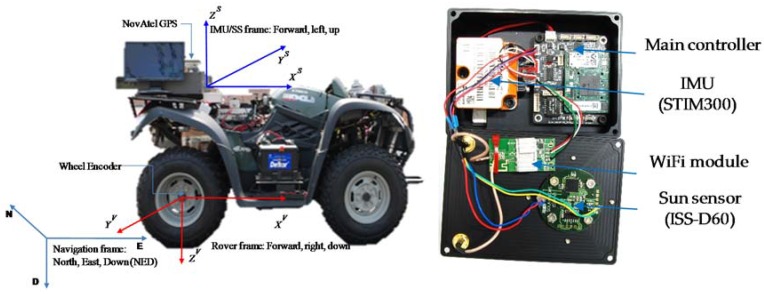
Left: Test rover. Right: Exploded view of the sensor module (*KAIROSS*) used for experiments.

We have developed a low cost, low weight and low power consumption navigation module called the Kitech Advanced Intelligent Rovers Sensor System *(KAIROSS)*, as shown in Figure 8 below. This prototype unit uses two navigation sensors, a ISS-D60 (Sun sensor, Solar MEMS Technologies, Sevilla, Spain) and STIM300 (IMU, Sensonor AS, Horten, Norway), based on MEMS technology. The Sun sensor is a PSD type sensor with two orthogonal axes and a wide field of view. The IMU consists of a 3-axis accelerometer, 3-axis gyros and 3-axis inclinometer, with onboard calibration and temperature compensation routines. The main parameters of *KAIROSS* and the sensors used in this work are given in Table 1.

In *KAIROSS*, we have used the OMAP-L138 processor from Texas Instruments (TI, Dallas, TX, USA) for on-board processing, which has a dual-core SoC, a ARM926EJ-S RISC MPU, and C674x DSP, with 8MB SPI flash memory for kernel image for stand-alone system, 10/100Mbps Ethernet peripheral for data and commands transfer, 3 UARTs for getting data from the sensors, USB2.0 for WiFi, I2C bus interface, and DMA, *etc.* This system has two separated processes. Getting data from the sensor is handled by the DSP processor so that it ensures exact time stamps; and on the ARM side, it runs the Linux kernel and handles communication with the main control center. *KAIROSS* can be operated with an external/internal battery pack and weighs only 270 grams.

The test rover used for the experiments is an MXU 500i (KYMCO, Spartanburg, SC, USA), an off-road rover that can be remotely controlled, and which was modified for autonomous mode capabilities as a UGV. The navigation system is installed on top of the test rover.

## 5. Experimental Set-up and Results Discussion

To verify our proposed algorithms, the experiments are conducted on an Earth-based test rover. The differences in terms of rover navigation between the Earth-based tests and actual rover (e.g., lunar rover) is that the Earth’s rotation angular velocity is about 27 times faster than that of the Moon, which results in a slower interval of Sun observations. The other major difference is the radius of the planet, which affects the positional accuracy as estimated on Earth-based test rovers. The Sun illumination and Sun appearance duration time may also be different than those on Earth [29]. We considered possible motion scenarios commonly encountered by planetary rovers. These include:

- Long time (e.g.,hours) stationary state: Planetary rovers remain stationary most of the time unless they are commanded to move forward or perform some task.
- Turn in place (*TiP*) motion: This is a very common motion scenario on a planet with many unforeseen obstacles. The rover has to avoid them by performing a turn-in-place motion to reach the goal with minimum energy and time.
  (i) *TiP* in large steps: Turn-in-place rotation test of the rover in steps of 90°, both clockwise (CW) and then counter-clockwise (CCW) was performed. This test was conducted to check the capabilities of the developed algorithm to track the changing absolute heading angle when the rover takes large turns to avoid obstacles on the planet surface.
  (ii) *TiP* in small steps: This is the *TiP* test in steps of 20°, to check the small angle change tracking of the algorithm.
  (iii) Continuous *TiP*: Sometimes the rover has to turn about continuously to change direction. This test verifies the algorithm’s ability to track absolute angle change during continuous turning of the rover from 0–360°, in CW and CCW rotations.
- With arbitrary attitude: Most of the time rovers are commanded to move forward a few centimeters and then look around, and take a curved path, with an arbitrary attitude, around any encountered obstacle or target rock, to take an appropriate action.

### 5.1. Long-Time Static Test

For Test (A), we kept the sensor module stationary for about 2.5 h at a fixed location (longitude: 126.841833° and latitude: 37.293353°) in a particular direction on 27 October 2014 and captured data from *KAIROSS*. The roll and pitch angles were 4.5° and 6°, respectively during this static test. The purpose of this experiment was to test the long term stability of heading estimation algorithms, both *EASI* and *q-Method*, using onboard sensors only. The position (longitude, latitude) of the rover was taken from *Google-Earth* and time/date was the onboard system time. In actual planetary rovers, however, the absolute position may be acquired from other sources.

**Figure 9:**
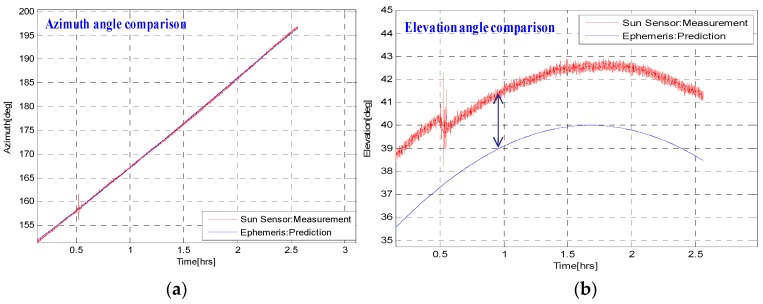
Azimuth angle (**a**) and Elevation angle (**b**) comparison of the *EASI* algorithm and Sun Ephemeris data.

**Figure 10:**
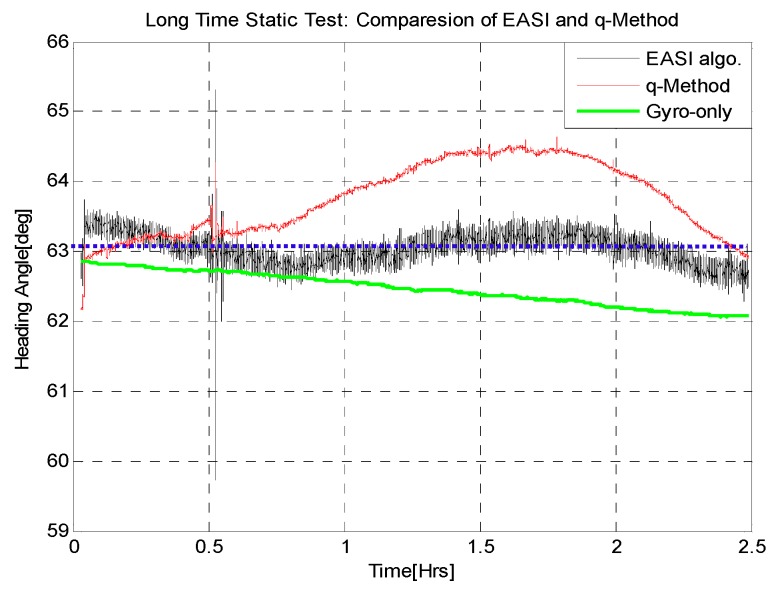
Rover heading angle estimation during the 2.5 h static test. Comparison of the *EASI* algorithm and the Optimization based *q-Method*.

The results of the long time stationary condition are shown in Figure 9 and Figure 10. In Figure 9, we compare the azimuth and elevation angles as predicted by the Sun Ephemeris data with that of the Sun sensor measurements, from 11:00 AM to 13:30 PM, a 2.5 h time duration. Both azimuth angles match as expected, but there is a fixed offset of about 3° in the elevation angles. This offset is due to the atmospheric effect as the Sun rays are refracted when entering the Earth’s atmosphere. This effect is called “*Albedo Effect*”; however, the Albedo Effect is absent in atmosphere-free planets, which is our intended application.

The final outputs of the two heading estimation algorithms are plotted in Figure 10. The absolute heading angle determined by the *EASI* algorithm is quite smooth with a mean error of 0.26°, standard deviation of 0.21° and a maximum error of less than 1°. However, the absolute heading estimate provided by the *q-Method* has an error of about 2°. This proves that the proposed algorithm (*EASI*) has better performance than the optimization based *q-Method* in this long term static test. In addition, the heading angle of *EASI* does not diverge, though it is noisier compared to the gyro-only solution, which inherently diverges as a result of the dead reckoning process. A quantitative comparison of the heading estimation algorithms is given in Table 2.

### 5.2. Turn-In-Place (TiP) Tests

The second motion scenario, *Turn-In-Place (TiP)* is a very important type of motion from a planetary rover’s perspective, as the rover has to take sharp turns to avoid unforeseen obstacles.

**Figure 11:**
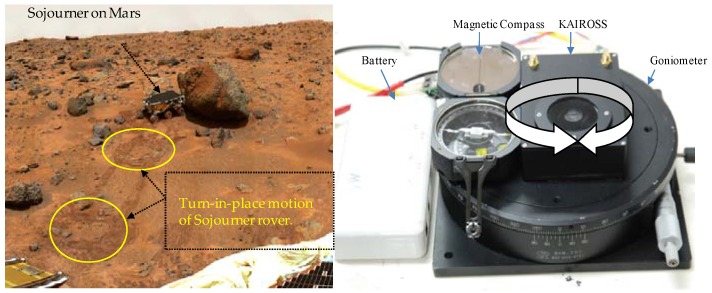
Turn-in-place motion: (**Left**) Actual Mars rover (Sojourner) took two *TiPs* before reaching the targeted rock [30]; (**Right**) Sensors set-up for *TiP* test (B), both CCW and CW rotation through 0–360°.

To test the *TiP* motion scenario (B), a sensor set up composed of *KAIROSS*, a Goniometer and a high precision magnetic compass was used, as shown in Figure 11.

**Figure 12:**
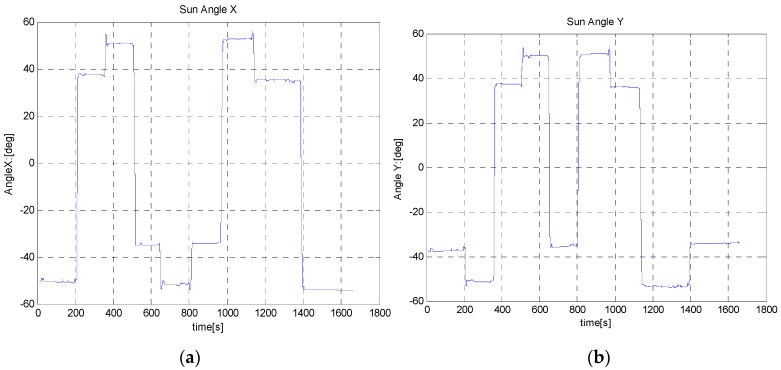
Sun sensor output during Test (B-i) with 90° rotation steps in the CW and CCW directions. (**a**) Angle-x is the angle between z-axis and projection of Sun ray on x-z plan; (**b**) Angle-y is the angle between z-axis and projection of Sun ray on y-z plan.

**Figure 13:**
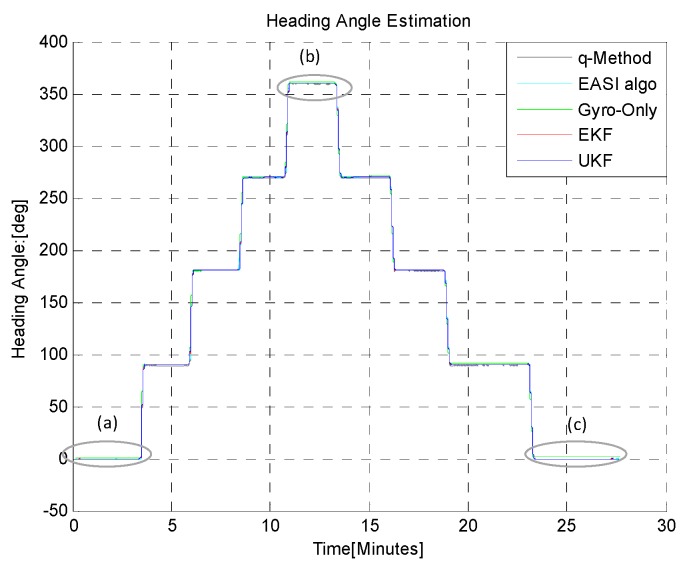
*TiP* with large steps (90°) in CW and CCW. Comparison of heading estimation algorithms.

**Figure 14:**
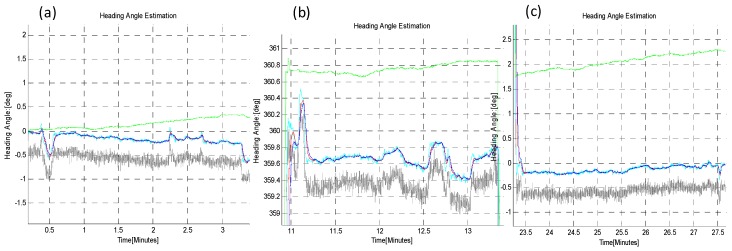
Zoom-in view of heading angle, (**a**) at start; (**b**) in middle and; (**c**) at the end of test.

In the first test (B-i), the sensor module was rotated through large steps of 90°, in CW and then CCW. For each step, the rover rested for about 2 min before performing the next rotation and completed a full 0~360° rotation and back. The output of the absolute heading estimation algorithms were compared against the reference value taken from the Goniometer readings, and the absolute heading angle measured by a very precise magnetic compass. The results of this test are shown in Figure 12, Figure 13 and Figure 14 and in Table 3.

**Figure 15:**
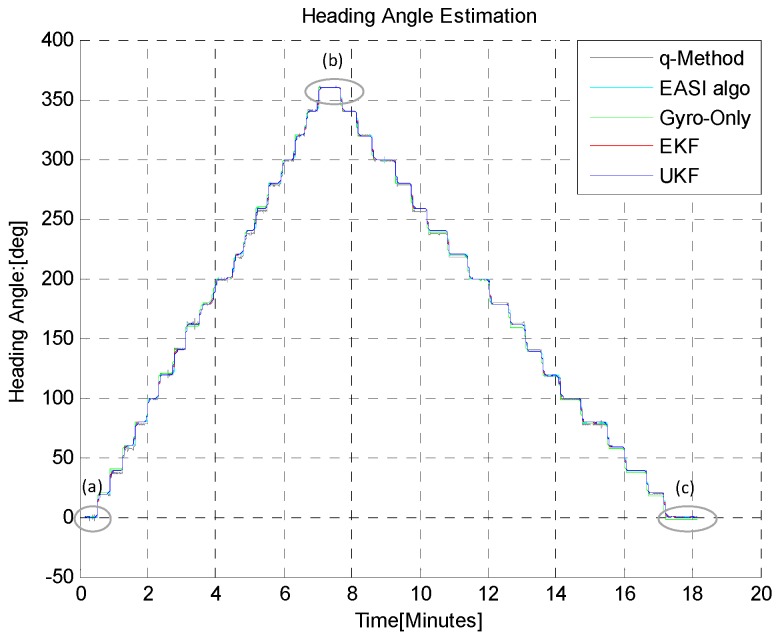
*TiP* with small steps (20°) in CW and CCW rotation. The EKF and UKF estimates end up with close to the initial heading angle but the gyro-only solution shows clear divergence, more than 2° at the end of this experiment.

**Figure 16:**
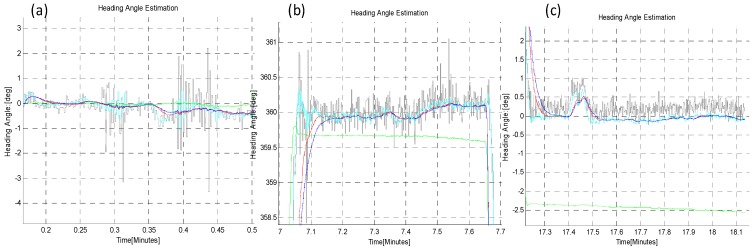
Zoom-in view in *TiP* test (B-ii), with small increments (20°), with both CW and CCW rotation through 0~360°, at the start (**a**); mid (**b**) and end **(c**) point of rotation. Clearly, the *q-Method* has large errors as compared to *EASI.*

In the second *TiP* test (B-ii), the sensor plate was rotated in short steps of 20°, to ensure that the developed algorithms can also reliably track small changes in absolute heading angle, while performing the turn-in-place rotation, as shown in Figure 15 and Figure 16. The zoom-in view (**a**,**b**,**c**) and statistical comparison are shown in Figure 16 and in Table 4. The *q-Method* has a large mean error and standard deviation as compared to the *EASI* algorithm. The *q-Method* does not track heading angle well in this quasi-dynamic test due to the dynamic motion, because it needs multiple measurements from the same position to perform an optimization operation.

**Figure 17:**
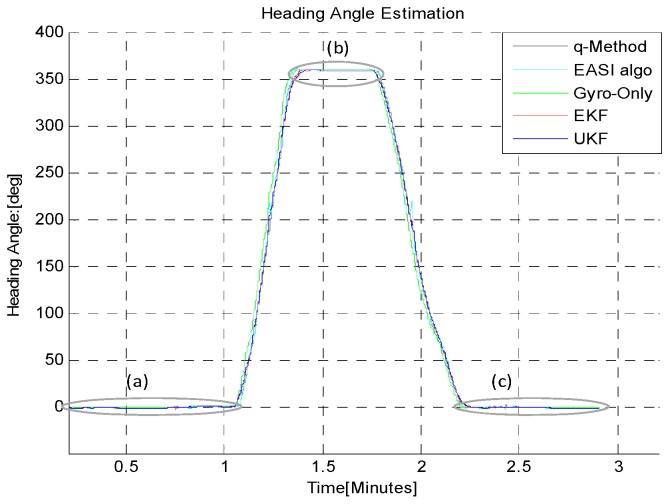
Continuous rotation (0~360°) in CW and CCW direction in the *TiP* test (B-iii). The *EASI* algorithm output gives a noisy but stable heading angle. EKF and UKF show similar performance in this short time continuous rotation experiment. The *q-Method* does not perform well, as compared to the *EASI*, due to sensor’s continuous motion.

**Figure 18:**
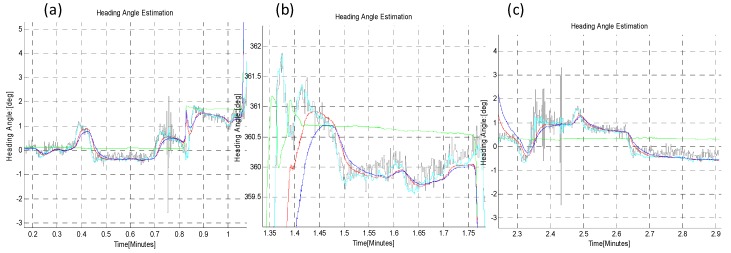
Zoom-in view of continuous Turn-in-place test (B-iii): Start (**a**); mid (**b**) and at the end (**c**) of rotation in CW and CW through 0~360° rotation. The *q-Method* heading estimate is noisy and has large errors due to the dynamic condition of the sensor.

In the third Turn-in-place test (B-iii), the sensor plate was rotated continuously in order to check the continuous tracking of absolute angle by both algorithms, as shown in Figure 17, Figure 18 and Table 5. This scenario of motion may sometimes be required to turn the rover back because of some large encountered obstacle, for example. The statistical comparison at the start (**a**), mid (**b**) and at the end (**c**) of this test shows that *EASI* algorithm has better performance against the *q-Method*, and a little better performance in UKF as compared to EKF is also observed in this application.

### 5.3. Long Time Dynamic Test

To test the algorithm’s performance under the dynamic condition of the rover (Test-C), the sensor module was fixed on a hand-driven cart and moved slowly (e.g., 2 cm/s.) in a stop-and-go fashion and with arbitrary roll, pitch and heading. At the start of the dynamic test, the sensor module was kept static for some time to capture the turn-on bias of the IMU. During motion, the Sun obstruction condition was also tested by blocking the Sun radiation for some time.

In this test, the sensor module was placed at a certain initial orientation, and it was arranged that the module would end up in same orientation at the end of this long dynamic test. Hence, the initial and final orientation angles were taken as reference values for error calculations. We drove our sensor module with different speeds and with an arbitrary attitude for about an hour. The motion was in a stop-and-go fashion in order to mimic the planetary rover’s motion. The integrated (or fused) results are expected to show superior performance in terms of accuracy and robustness in comparison to a single navigation sensor.

The EKF and UKF were compared for accuracy and robustness by simulating a scenario in which the Sun radiations were obstructed for some reason, such as the rover being under the shadow of a big rock, or when the Sun sensor is occluded by the rover’s own shadow. In that case the Sun’s radiation was below the critical value of 300 W/m^2^. In Sun obstruction situations, the absolute heading information from *EASI* or *q-Method* is not available to update the EKF/UKF.

**Figure 19:**
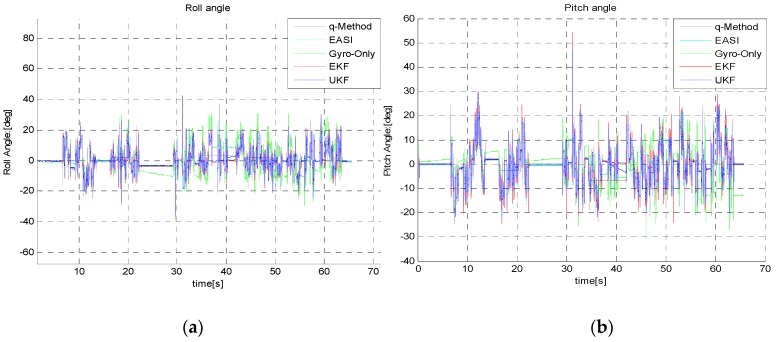
(**a**) Roll angle and (**b**) pitch angle estimation during the long dynamic test.

**Figure 20:**
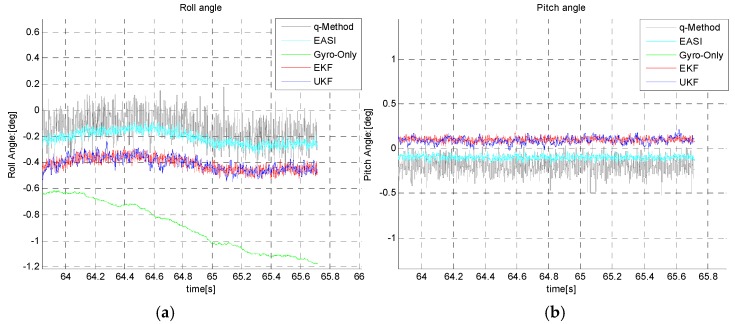
End trajectory Roll angle (**a**) and pitch angle (**b**) estimation during long time dynamic test.

Roll and pitch angles propagated by the gyro-only method show clear divergence (Figure 19), whereas the EKF/UKF has almost the same performance, as is evident from the end-trajectory zoomed-in views (Figure 20). The numerical comparison of roll/pitch is given in Table 6, below.

**Figure 21:**
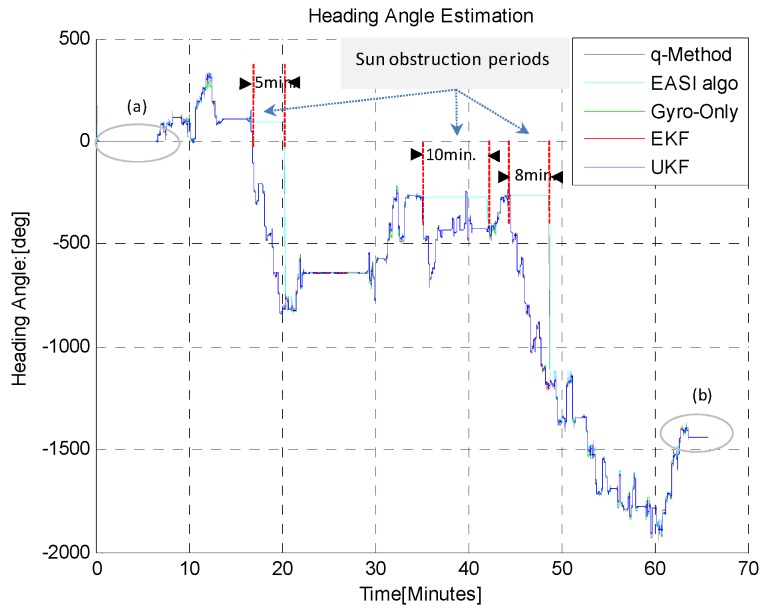
Heading angle (unwrapped) estimated by different algorithms during the one hour dynamic test.

The estimated heading angle provided by different algorithms is given in Figure 21. There were Sun obstructions periods of different time durations during this long-time dynamic test, in order to check the capability of the estimators, EKF/UKF during Sun obstruction periods.

**Figure 22:**
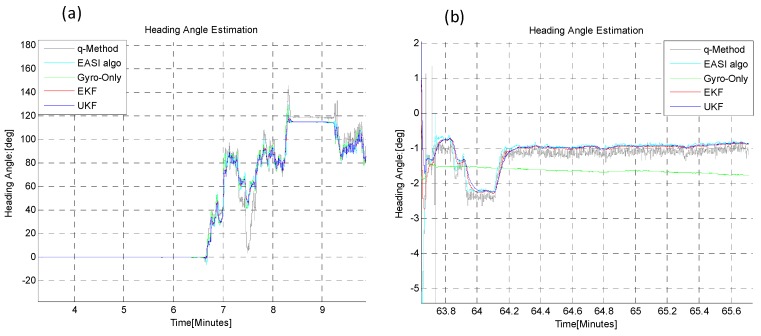
Zoom-in view of Start (**a**) and End (**b**) heading angle estimation in the dynamic test of 1 h. The *q-Method* is not in agreement with the other algorithms in the dynamic condition. However, in the static condition (e.g., before 6.5 min and after 63.8 min), the *q-Method* also performs nearly as well, as expected.

**Figure 23:**
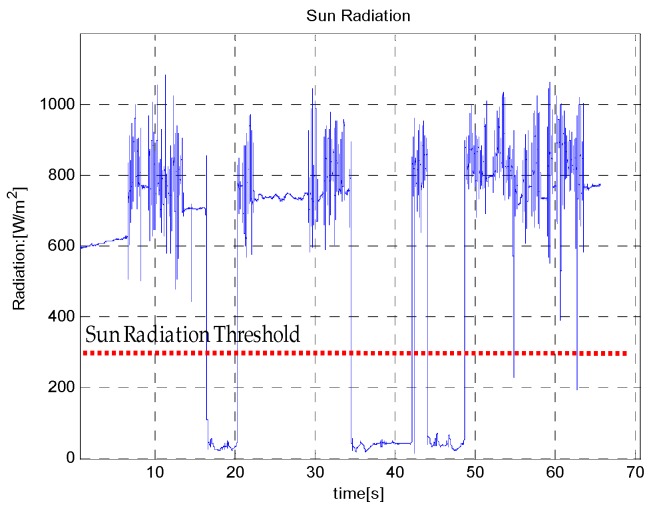
Sun radiation during the long-time dynamic test. Note that below a threshold of 300 W/m^2^, the sensor output is not reliable for absolute heading calculation.

Three Sun-obstruction periods of different time lengths (5, 10 and 8 min) were considered during the motion, to check the robustness of the heading estimation output of the EKF/UKF algorithms. It was noted that during Sun obstruction periods, neither the *EASI* nor the *q-Method* work because of the Sun sensor limitations. However, the fused data of IMU and *EASI* (*i.e.*, the EKF/UKF) provided heading angle tracking even during the Sun obstruction periods. The zoom-in view of the start and end heading angle is given in Figure 22. The Sun radiation data with threshold for this experiment is shown in Figure 23. The quantitative comparison of heading estimation algorithms is given in Table 7.

The comparison of *EASI* and *q-Method* shows that in the static condition, both absolute heading estimation algorithms work well as expected, but in the dynamic case, the *q-Method* fails to estimate heading reliably, whereas the *EASI* has good performance both in the static and in the dynamic situation too. The reason is that *EASI* is a *one-shot* algorithm whereas the *q-Method* requires the collection of a number of measurements before applying some kind of optimization method.

**Figure 24:**
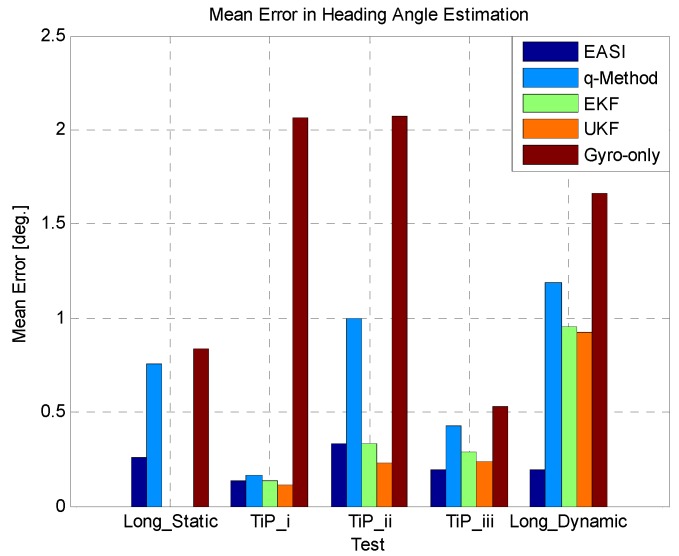
Mean error in all five tests.

**Figure 25:**
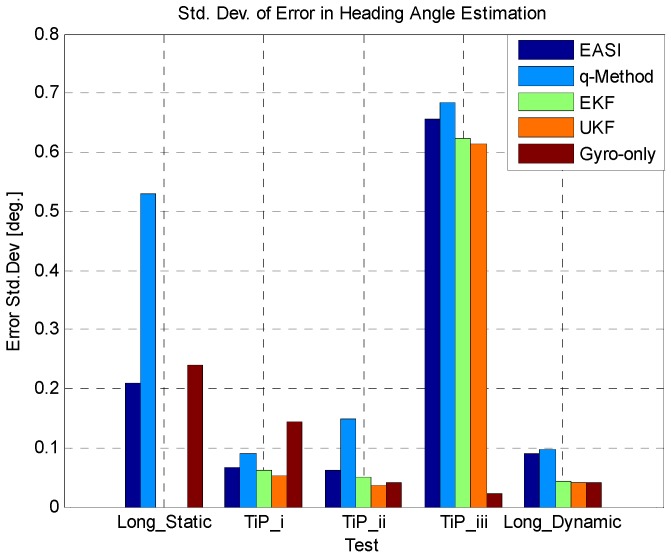
Standard deviation of error in all five tests.

In Figure 24 and Figure 25, we have summarized the statistical results of all five tests conducted in this study, providing the mean error and standard deviation of all the algorithms compared in each test.

Both the qualitative and quantitative results confirm that the developed algorithm, *EASI* outperforms the conventional optimization-based *q-Method*, especially in the dynamic condition. It was also demonstrated in this work that multisensory fusion has higher accuracy and robustness than the individual sensor/methods, in estimating attitude for the planetary test rover.

### 5.4. Planetary Rover’s Localization

**Figure 26:**
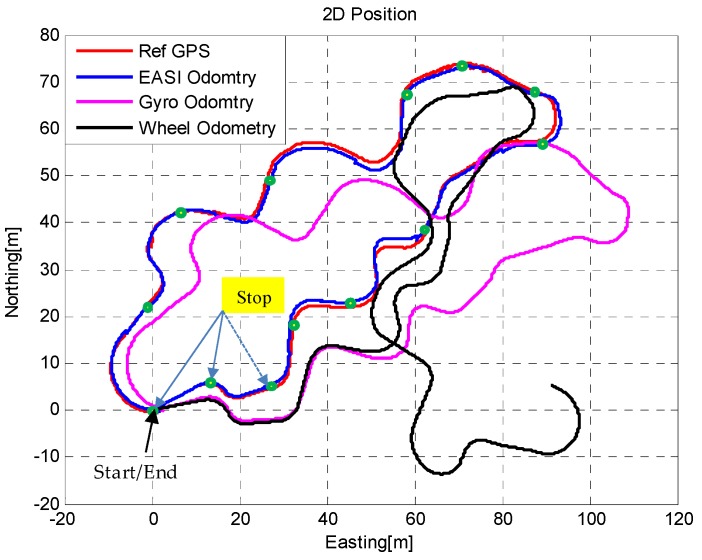
2D localization plot: Comparison of position estimations using different localization techniques for the planetary rover.

For completeness, we also developed an integrated position estimation algorithm for the planetary rover, shown in Figure 26. For comparison, three localization methods were considered: conventional *wheel-odometry* as for ground robots, *gyro-odometry* and *EASI-odometry*. In *wheel-odometry*, the forward position change is calculated for every motion from pre-calibrated encoder data, and heading angle is calculated from steering wheel encoder data. However, this kind of motion estimation is only valid over a limited period of time and on flat surfaces. Depending on surface roughness, this method is not reliable for long time operations.

However, the performance of *wheel-odometry* can be enhanced by including the gyro data in the attitude calculation. Instead of using only heading angle from the steering wheel encoder, in *gyro-odometry*, 3D attitude angles are used to propagate wheel position data. This type of position estimation has good performance as compared to wheel encoders only, but still depends on the accuracy of the gyros used. During long time operations, *gyro-odometry* also suffers from drift and position estimates become no longer usable, and cross-track errors especially have increased error in estimating heading angle.

**Figure 27:**
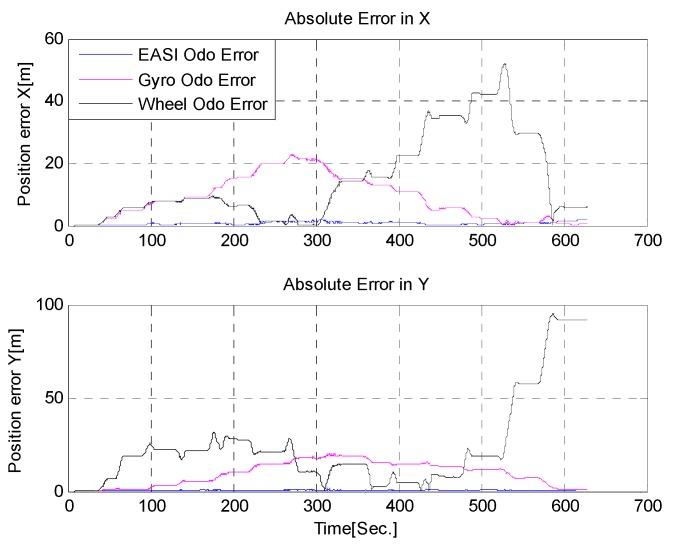
Absolute error in position estimation.

To reduce errors in *gyro-odometry*, we must compensate attitude errors by some absolute means, e.g., from the inclinometer and Sun sensor data used in this work. By combining the Sun sensor and IMU, called *EASI-odometry*, the errors in localization of the rover are also greatly reduced. The localization estimates were compared with the high-performance NovAtel GPS module with 5 m accuracy. The absolute error in position estimates are shown in Figure 27 below. The quantitative comparison of the three position estimation methods is given in Table 8.

The quantitative comparison of position estimates shows that the position errors of the *EASI-odometry* were less than 3m when compared with a NovAtel GPS module in this 10 min and about 200 m run.

## 6. Conclusions

In this work, we have presented methods for estimating attitude and position using different algorithms which only employ on-board low cost, low power MEMS sensors. Two absolute heading estimation algorithms were developed and compared for accuracy and reliability. The *EASI* algorithm outperformed the traditionally used optimization-based *q-Method* in rover’s in-motion condition. However, in static tests, both algorithms exhibited comparable performance. The drawback of the absolute heading angle determination algorithms is that their output is noisier and the Sun sensor does not produce any measurements below a certain Sun light radiation (<300 W/m^2^). At the same time, the gyro-only attitude determination has an inherent limitation during long time operations, as the attitude angles start drifting after some time.

To cope with this issue, the output of the absolute attitude estimation algorithm was fused with the relative attitude sensor, *i.e.*, MEMS gyros, to reduce error drift in attitude with the passage of time. The fused attitude solution provides a reliable heading angle in the event of Sun radiation blockage and has better accuracy as compared to absolute heading algorithms alone. Furthermore, the fused attitude is non-drifting and less noisy as compared to the gyro-only or *EASI/q-Methods*.

For the sensor fusion in this nonlinear problem, we compared two commonly known filters, the Extended Kalman filter and Unscented Kalman filter. The UKF has a little better performance than EKF in this application; however, both filters have error <1°, in heading angle estimation. For completeness, we also compared the localization results of three algorithms, and it was shown that when the localization algorithm was integrated with the *EASI* algorithm, the errors in the position were also reduced to less than 3 m in a 200 m run, which is about 1.5% of the total distance travelled. As a future research work, the absolute heading estimation algorithm will be integrated with other localization systems, e.g., visual odometry, DEM matching, *etc.*

## Figures and Tables

**Table 1:** Sensor parameters in *KAIROSS*.

Sensors	Parameters	Wt. (g)	Pwr. (w)@5V	Size (cm)
Sun Sensor (ISS-D60)	Accuracy:0.4°;2-axis; FOV:120° × 120°	100	0.165	r = 4, h = 2.1
MEMS IMU(STIM 300)	Bias stability: 0.5 (°/h); ARW: 0.5(°/√h)	55	2	3.9 × 4.5 × 2.2
*KAIROSS*	-	270	3.2 (Load)	11 × 8 × 4.3

**Table 2:** Statistical comparison of heading estimation algorithms.

	Error (°)	Mean Error	Std. dev.	Max. Error	RMS Error
Algo.					
*q-Method*		0.76	0.53	1.8	0.93
*EASI *		0.26	0.21	0.7	0.34
Gyro-only		0.84	0.24	0.5	1.16

**Table 3:** Statistical comparison of heading estimation algorithms in the 1st *TiP* test (B-i).

	Error (°)	Start (0°)		Mid (360°)			End (0°)	
Algo.		Mean	Std. dev.	Mean	Std. dev.	Error	Mean	Std. dev.
*EASI*		−0.158	0.095	359.65	0.112	0.348	−0.134	0.067
*q-Method*		−0.163	0.117	359.74	0.125	0.256	−0.165	0.090
EKF		−0.158	0.090	359.65	0.105	0.349	−0.134	0.063
UKF		−0.154	0.085	359.65	0.102	0.348	−0.113	0.052
Gyro-only		0.152	0.103	360.75	0.055	0.759	2.062	0.145

**Table 4:** Statistical comparison of heading estimation algorithms in *TiP* test (B-ii).

	Error (°)	Start (0°)		Mid (360°)			End (0°)	
Algo.		Mean	Std. dev.	Mean	Std. dev.	Error	Mean	Std. dev.
*EASI*		0.241	0.265	360.410	0.093	0.414	0.335	0.063
*q-Method*		0.605	0.625	360.860	0.180	0.862	0.995	0.148
EKF		0.255	0.192	360.400	0.077	0.405	0.333	0.049
UKF		0.179	0.278	360.400	0.073	0.400	0.231	0.038
Gyro-only		0.324	0.334	360.040	0.022	0.049	-2.073	0.040

**Table 5:** Statistical comparison of heading estimation algorithms in the continuous *Tip* test.

	Error (°)	Start (0°)		Mid (360°)		End (0°)	
Algo.		Mean	Std. dev.	Mean	Std. dev.	Mean	Std. dev.
*EASI*		0.332	0.676	360.111	0.411	0.191	0.657
*q-Method*		0.651	0.694	360.297	0.414	0.427	0.685
EKF		0.326	0.659	360.101	0.396	0.285	0.624
UKF		0.301	0.651	360.026	0.307	0.234	0.634
Gyro-only		0.495	0.698	360.632	0.055	0.532	0.022

**Table 6:** Statistical comparison of estimated tilt angles in the long-time dynamic test.

	Error (°)	Roll				Pitch			
		Stat		End		Start		End	
Algo.		Mean	Std. dev.	Mean	Std. dev.	Mean	Std. dev.	Mean	Std. dev.
*EASI*		−0.042	0.026	−0.111	0.055	−1.192	0.021	−1.097	0.021
*q-Method*		−0.061	0.088	−0.238	0.105	−1.602	0.082	−1.689	0.089
EKF		−0.268	0.029	−0.413	0.051	−0.982	0.025	−0.898	0.022
UKF		0.269	−0.036	−0.413	0.055	−0.980	0.038	−0.909	0.037
Gyro-only		−0.542	0.057	−0.937	0.163	0.050	0.041	−13.875	0.066

**Table 7:** Statistical comparison of heading estimation algorithms in the dynamic test (only end-trajectory errors are compared).

	Error (°)	Mean	Std. Dev.	Max. Error
Algo.				
*EASI*		−0.912	0.090	1.091
*q-Method*		−1.186	0.096	1.7105
EKF		−0.954	0.044	1.067
UKF		−0.928	0.041	1.017
Gyro-only		−1.664	0.042	1.755

**Table 8:** Statistical comparison of different localization methods.

	Error (m)	Abs. Error (max)		2D Abs. Error	RMS Error		2D RMS Error
Algo.		x	y	$\sqrt{x^{2}+y^{2}}$	x	y	$\sqrt{x^{2}+y^{2}}$
Wheel Odo.		52.1	95.3	108.6	20.2	34.3	39.8
Gyro Odo.		22.8	20.5	30.7	11.3	11.6	16.2
*EASI Odo.*		1.8	1.6	2.4	0.9	0.6	1.1
